# Impact of test, vaccinate and remove protocol on home ranges and nightly movements of badgers in a medium density population

**DOI:** 10.1038/s41598-023-28620-1

**Published:** 2023-02-14

**Authors:** Sophie H. A. Redpath, Nikki J. Marks, Fraser D. Menzies, Maria J. H. O’Hagan, Rory P. Wilson, Sinéad Smith, Elizabeth A. Magowan, David W. McClune, Shane F. Collins, Carl M. McCormick, D. Michael Scantlebury

**Affiliations:** 1 School of Biological Sciences, Queens’ University Belfast, 19 Chlorine Gardens, Belfast, BT9 5DL Northern Ireland; 2 Department of Agriculture, Environment and Rural Affairs, Veterinary Epidemiology Unit, Belfast, BT4 3SB Northern Ireland; 3 Department of Biological Sciences, Swansea University, Singleton Park, Swansea, SA2 8PP Wales; 4 Present Address: Veterinary Sciences Division, Agri-Food and Biosciences Institute, Belfast, BT4 3SD Northern Ireland

**Keywords:** Animal behaviour, Behavioural ecology, Ecophysiology

## Abstract

In the British Isles, the European badger (*Meles meles*) is thought to be the primary wildlife reservoir of bovine tuberculosis (bTB), an endemic disease in cattle. Test, vaccinate or remove (‘TVR’) of bTB test-positive badgers, has been suggested to be a potentially useful protocol to reduce bTB incidence in cattle. However, the practice of removing or culling badgers is controversial both for ethical reasons and because there is no consistent observed effect on bTB levels in cattle. While removing badgers reduces population density, it may also result in disruption of their social behaviour, increase their ranging, and lead to greater intra- and inter-species bTB transmission. This effect has been recorded in high badger density areas, such as in southwest England. However, little is known about how TVR affects the behaviour and movement of badgers within a medium density population, such as those that occur in Northern Ireland (NI), which the current study aimed to examine. During 2014–2017, badger ranging behaviours were examined prior to and during a TVR protocol in NI. Nightly distances travelled by 38 individuals were determined using Global Positioning System (GPS) measurements of animal tracks and GPS-enhanced dead-reckoned tracks. The latter was calculated using GPS, tri-axial accelerometer and tri-axial magnetometer data loggers attached to animals. Home range and core home range size were measured using 95% and 50% autocorrelated kernel density estimates, respectively, based on location fixes. TVR was not associated with measured increases in either distances travelled per night (mean = 3.31 ± 2.64 km) or home range size (95% mean = 1.56 ± 0.62 km^2^, 50% mean = 0.39 ± 0.62 km^2^) over the four years of study. However, following trapping, mean distances travelled per night increased by up to 44% eight days post capture. Findings differ from those observed in higher density badger populations in England, in which badger ranging increased following culling. Whilst we did not assess behaviours of individual badgers, possible reasons why no differences in home range size were observed include higher inherent ‘social fluidity’ in Irish populations whereby movements are less restricted by habitat saturation and/or that the numbers removed did not reach a threshold that might induce increases in ranging behaviour. Nevertheless, short-term behavioural disruption from trapping was observed, which led to significant increases in the movements of individual animals within their home range. Whether or not TVR may alter badger behaviours remains to be seen, but it would be better to utilise solutions such as oral vaccination of badgers and/or cattle as well as increased biosecurity to limit bTB transmission, which may be less likely to cause interference and thereby reduce the likelihood of bTB transmission.

## Introduction

Bovine tuberculosis (bTB) is a zoonotic disease that is prevalent in cattle which threatens animal health, farm productivity and commercial earnings in affected countries^1,2^. *Mycobacterium bovis,* the main causative agent of bTB, is capable of infecting a wide range of species including companion animals^3,4^, wild mammals^5,6^, livestock^7^ and humans^8^. Cattle are considered to be the primary maintenance host of the disease in the United Kingdom (UK) with European badgers (*Meles meles*) acting as a wildlife reservoir that contributes to disease transmission^6,7,9^. Although most cases of bTB in cattle are understood to result from cattle-cattle transmission^13,22–25^, endemic infection of bTB within wild populations of badgers occurs^26^. Some studies have reported cattle-to-badger transmission^22,31^ as being more likely to occur than badger-to-cattle transmission, with identical bTB strains have been discovered in local cattle and badger populations^22,27^. However, the direction and frequency of disease transmission between cattle and badgers in many instances have so far not been established^6,10^. Despite efforts to control and eradicate bTB, disease prevalence in cattle herds is persistently high in some areas, such as the southwest of England (11.52%) and the County Down region of Northern Ireland (NI) (9.47%)^11,12^. Transmission of *M. bovis* between cattle and badgers may occur by direct or indirect contact^2,10,13^, which can be influenced by cattle, and/or badger density, and vary between areas and habitats^7,9^. In England, the high prevalence of bTB in cattle in some areas co-occurs with high badger density, which can be as high as 25 badgers per km^2^^14–16^. Badger density (individuals per km^2^) in randomly sampled areas of England and Wales (mean = 3.29 per km^2^, range = 0.26–5.98 per km^2^^17^) is comparable to the badger density recorded in NI (mean = 2.78 per km^2^, range = 0.7 and 3.88 badgers per km^2^^18,19^). However, despite the smaller average cattle herd size in NI^20,21^, cattle density is high, which may lead to increased contact rates between badgers and cattle. The prevalence of bTB in cattle herds during 2022 was observed to be as high in NI (10.49% of herds containing at least one reactor^12^) as in some high bTB areas in England and Wales (9.6–9.9%^11^). Several factors may influence the transmission of bTB between individuals, such as host age, body condition, and season^15^. Less is known, however, how socio-spatial behaviour, such as ranging, may affect bTB transmission rates between cattle and badgers, especially in areas with lower badger, but high cattle density.

Proactive badger culling (removal of up to 70% of individuals in an attempt to keep population size at a specified minimum^22,23^) has been undertaken in various locations in efforts to reduce the incidence of bTB in cattle in England (e.g., Gloucestershire and Somerset, 2013–2017, and elsewhere, 2021^22^) and the Republic of Ireland (ROI) (e.g., Cork, Donegal, Kilkenny and Monaghan, 1997–2002)^23–26^. As some studies show a positive association between badger population density and the risk of a cattle herd becoming infected^27,28^, culling badgers has been suggested as a method to reduce badger density and thereby reduce potential contact rates between badgers and cattle^13,24^. However, the effects of culling are complex. In the ‘Randomised Badger Culling Trial’ in England, there was an increased bTB risk of infection in cattle herds up to 2 km from the core badger culling area, which lasted two years after the cull^26,29^. Culling was suggested to disrupt territorial behaviour in badgers, creating a ‘vacuum’ in culled areas, in which neighbouring badgers increased their home range size to envelop the vacant territory^30,31^—the ‘perturbation’ effect^32^. Perturbation alters badger social group structure, which can change the spatial organization^30^, leading to increased social group overlap, home range size and inter-group movements, for up to several years after culling has ceased^29,30,33,34^. Whilst wider ranging behaviour is typically observed in heavier individuals and with male badgers^35^, an increase in ranging across the population is likely to increase the risk of contact between infected and non-infected individuals^33^. However, the perturbation effect has not been reported in ROI^23,25^, possibly due to differences in culling intensity^36^, habitat and/or badger density^14^.

Various approaches have been used to determine the intricacies of badger movements and behaviour including direct observation^37^, VHF radio telemetry^30,34,38^, aRFID (radio frequency identification)^39,40^, camera surveillance^2^, the spool-and-line technique^37^, magnetic localisation^38^, and use of Global Positioning System (GPS) loggers^24,34^. Most of these methods are limited in the detail they can provide, and in some cases, the presence of field researchers may cause stress-associated changes in behaviour^41,42^. An alternative method for determining badger movement tracks is GPS-enhanced ‘dead-reckoning’^43–45^. Whilst this method still requires animals to be captured, it does enable fine-scale animal movement paths to be elucidated using data from animal-borne tri-axial accelerometers, tri-axial magnetometers and GPS loggers, which together can provide higher resolution data than the use of GPS devices alone^43–46^. The aims of this study were to determine the movements and home range sizes of badgers within a medium badger density area in NI that has a relatively high prevalence of bTB in cattle, both before and during an ongoing ‘test, vaccinate or remove’, ‘TVR’, protocol as an alternative to proactive badger culling. Based on previous findings in ROI^23,47^, we hypothesised that TVR may not necessarily be associated with long-term increases in home range size or ranging. However, we postulated that disturbances caused by overnight trapping procedures could well be associated with short-term effects on ranging behaviours in the days following capture, in a previously undisturbed population^41,42^. Finally, we hypothesised that ranging would be biased towards male badgers^47–49^.

## Methods

### Study area

**Figure 1:**
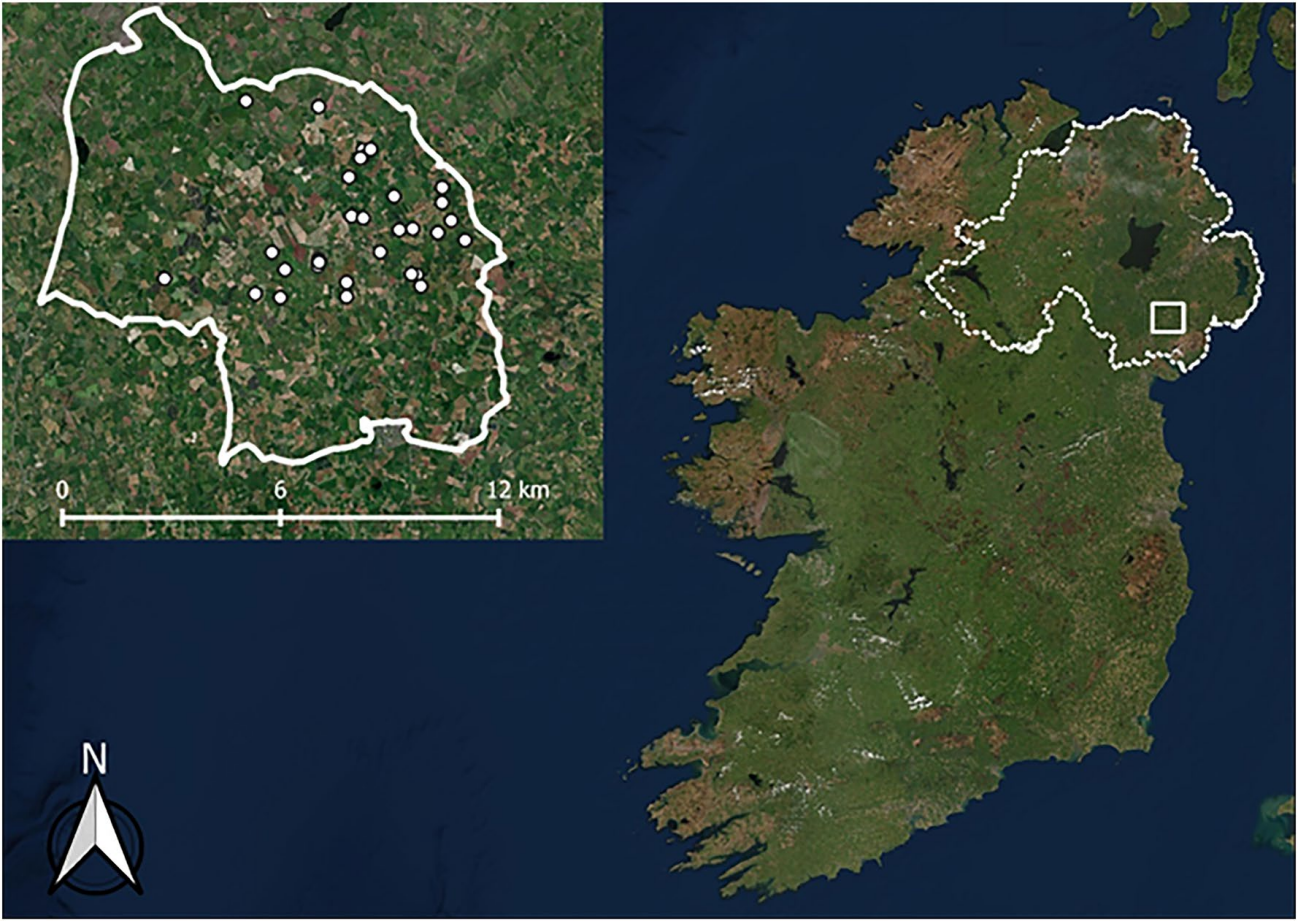
Map of study area in Northern Ireland (right) as shown by inset (left) created using QGIS version 3.8.1 (basemap satellite imagery ERSI World Imagery map) (https://www.qgis.org/en/site/ and https://www.arcgis.com/home/item.html?id=10df2279f9684e4a9f6a7f08febac2a9). Location of the study area relative to the whole of Ireland is shown in by a white border. White dots denote trapping locations.

The study area was located within a 100 km^2^ region in County Down, NI, near the town of Banbridge. It comprised primarily pasture and arable land that was enclosed within the boundaries of the A1 main road and the river Bann (Fig. 1). The study utilised animals that were captured as part of the TVR project^50–52^. An initial survey of the area, conducted in 2012, established the locations of badger “setts” (underground burrows where badgers reside^53^) in this region of NI^54^. The study area was chosen because it had a relatively high level of cattle bTB herd breakdowns along with relatively high cattle and badger sett densities compared to other areas of NI^52,54^. GPS data from badgers that were captured from June to October 2014 were collected. During this time, no removal of badgers took place (see details below regarding logger deployment). During subsequent years, between July to October in 2015, 2016 and 2017, bTB test-negative badgers were captured and fitted with GPS collars (recording positional fixes from July-February). No badgers were captured between November and May during each of these years due to legal restrictions preventing interference to reproductive females and offspring^52^. As climatic variables can influence badger ranging behaviour^14,18^, weather information data (mean daily temperature, total precipitation), obtained from the local UK Meteorological Office weather station (Katesbridge, Co. Down) were included in statistical analyses.

### Badger trapping

Different locations within the study site were trapped in a 3-week cycle, with the first week of each area devoted to surveying and placing traps around active setts^51,52^. Traps were then pre-baited with peanuts during the second week, and during the third week, they were set. This was done silently by a person on foot for four consecutive nights. To maximise the likelihood of recapturing an animal with a movement-sensitive daily diary ‘DD’ logger (see below for a description of loggers), DD loggers were deployed on the boundaries of one trapping area to the next, so that an animal would be likely to be captured during the trapping cycle of a subsequent area. Note when badgers were recaptured, the DD loggers were removed but the GPS and collar remained on the animal. Further details on the trapping process are available in the standard operating procedures supplemental material of^51^. Captured badgers were sedated using a ketamine-medetomidine-butorphanol combination (0.25 mL/kg dosage) by intramuscular injection while they were in the cage^50–52^. The sex, body mass (kg), body size (nose tip to tail, cm) and head size (nose to back of head, cm) of each individual were recorded^50–52^. As part of the TVR procedure, from 2015 onwards, captured badgers were tested in the field for *M. bovis* antibodies using a Dual Path Platform (DPP) VetTB test (Chembio Diagnostic Systems Inc., Medford, NY, USA)^50–52^. A positive test was denoted by 1 or 2 bands appearing on the test corresponding with *M. bovis* antigens MPB83 and ESAT-6/CFP-10, respectively. Individuals that tested positive were subsequently euthanised using intravenous pentobarbitone. Badger removal varied with year, (n = 56, 11, 22, 19 individuals removed during years 2, 3, 4, 5 respectively, out of a total population of c. 560 individuals, equating to 0.27 badgers/km^2^/year)^51,52^. This compares with removal rates of 0.57 badger/km^2^/year in the ‘Four Areas trial’ in Ireland^23^, and 1.8 badgers/km^2^/year in the ‘Randomised Badger Culling Trial’ in England^26,32^), resulting in fewer test-positive individuals being removed than previous studies^36,52^. Individuals that tested negative were vaccinated with BadgerBCG (during 2014–2016) or BCG Sofia (during 2017)^55^, and thereafter, microchipped and fitted with a neck collar and released^51,52,56^. Note, that a single vaccination of badgers with BCG Sofia did not result in “measurable detection of antibodies against MPB83 using Dual Path Platform (DPP) VetTB”^57^. The collar contained a GPS logger (Tellus Light, Followit, Sweden) to which a DD logger (Wildbyte Technologies, Swansea, UK) was affixed (below)^45,46,56^. During anaesthesia, samples of tracheal aspirate, nasopharyngeal secretions and blood were collected from each individual. These samples were subsequently used to determine *M. bovis* infection status by culture, interferon gamma (IFN-γ) blood testing and DPP testing^50–52,57^. After sampling, badgers were placed inside the trap where they were captured, and, following natural recovery (or anaesthesia reversal using atipamezole (Antisedan, Zoetis UK Ltd.) if natural recovery took over 1 h) were released at the site of capture^52^. Traps were then re-baited and re-set to enable further capture of animals on subsequent nights. Badgers were recaptured between 2 and 20 days post initial capture, at which point the DD logger was removed from the collar and the individuals were released. The GPS collar remained on the animal for up to 8 months, which enabled longer-term GPS data to be collected.

### Collar attachment and logger deployment

Some of the trapped badgers were equipped with a GPS collar. The decision as to whether a certain individual was chosen to have a collar was dependent on whether they tested DPP test-negative in the field (in 2015–2017), whether they were adults weighing more than 8 kg, and whether they had a head diameter 1 cm larger than their neck diameter (to ensure the collar could not easily slip off when they were released)^58^. In the event, when badgers were captured for the purposes of deploying DD loggers, all individuals were heavier than 8 kg. Therefore, it was not the case that certain individuals were excluded from having a DD tag (e.g., those with poorer body condition). We attempted to equip one male and one female with a collar containing a DD from each social group. Collars had a DD logger affixed using two crossed cable ties (30 cm long, 4.8 mm wide) and Tesa® tape (No. 4651; tesa AG, Hamburg, Germany)^56^. DD loggers were encapsulated within 3D printed styrene plastic cases, each with a 3.6 V battery (1/2 AA 3.6 V 1200 mAh Lithium Thionyl Chloride, Saft, Levallois-Perret, France), which was secured to the collar that contained the GPS (Tellus Light, Followit, Sweden) (total weight c. 270 g). Daily Diary loggers were attached to the subject such that the X-axis corresponded to the ‘surge’ motion (front-back acceleration), the Z-axis with ‘sway’ (left–right acceleration) and the Y-axis with ‘heave’ (up-down acceleration)^46^. Device magnetometers were calibrated by rotating them through 360° to correct for magnetic hard and soft iron distortion^59^. The GPS units recorded position fixes until the battery power was spent (up to 273 days post-trapping) and were programmed to record a locational fix once per hour between 21:00 and 04:00^47,52^, which corresponded to the period when badgers were likely to be most active^45^. Collars transmitted between one and eight fixes per night, dependent on GPS signal availability (for example, fixes were unlikely to be transmitted when badgers were underground^60^). Collars were positioned so that the accelerometer casing was on the side of the neck, the GPS battery on the bottom and the GPS receiver on the top.

### Data analyses

Of the 46 individuals that were captured and equipped with GPS and DD loggers, three of the GPS loggers and three of the DD loggers had hardware failures. A further two of the DDs were not retrieved after initial deployment. Therefore, data were available for a total of 38 badgers. This was approximately 6.79% of the population within the study area (n = c.560,^50–52^) and was a higher sample size than the minimum recommendation of 20–30 individuals recommended for home range analysis^61^. In terms of years of the study, data were available for 10 badgers in 2014 (five males and five females), nine badgers in 2015 (three males and six females), 11 badgers in 2016 (seven males and four females) and eight badgers in 2017 (six males and two females).

#### Home range size calculation

The home range sizes of individual badgers were determined using the recorded GPS data (Followit GEO, Lindesberg, Sweden) using the R packages adehabitatHR^62^ and ctmm^63^. Home ranges were visualised using geographical information system software (QGIS 3.8^64^). ‘Total’ home range areas, based on available GPS fixes, were identified using 95% autocorrelated kernel density estimates (AKDE), and ‘core’ home range areas were identified using 50% AKDE^65,66^. Autocorrelated kernel density estimates were used to account for irregular sampling frequency and reduced tracking data^67^. In this case, data were missing because GPS fix loss occurred due to badgers’ fossorial (burrowing) nature^60^. Autocorrelated kernel density estimate isopleths were defined as 95% for total home range and 50% for core areas to enable results from the current study to be compared with those of previous studies^68–70^. The centre point of 50% AKDE polygons were processed in QGIS v.3.16.0^64^, and the Euclidean distance travelled between them was determined with the software measurement tool to determine the distance an individual moved between core areas^71^. Minimum Convex Polygon estimates (95% MCP) and Local Convex Hull estimates (95% LoCoH) were used in addition to AKDE to measure total home range size^24,70,72^. The three methods differ in the estimations provided, with LoCoH providing values that are suggested to be more accurate when compared to MCP estimates but approximately 50% smaller due to lessened sensitivity toward outlier points, thereby excluding “unused” areas^70^. However, MCP estimates are commonly cited and are suggested to be more accurate than LoCoH estimates when there are few GPS fixes^73^. Due to a lack of standardisation when estimating home ranges, all three methods (MCP, LoCoH and AKDE) were used to calculate home ranges in order to enable results in the current study to be compared with those of previous studies^13,24,34,47,74^.

#### Nightly distance travelled determinations

The distance badgers travelled per night (km) were calculated using two different estimates. Minimum ‘GPS distance’ travelled per night was measured as the Euclidean distances between sequential GPS fixes using the R package ‘geosphere’^75^. ‘Dead reckoned’ coordinates were calculated using ‘Daily Diary Multiple Trace’, ‘DDMT’ software^43^ (see^76^ for discussion of dead reckoning procedure) which uses accelerometery data to calculate animal speed and magnetometry data to determine heading, whilst animals are known to be travelling, in order to create a high-resolution record of the animal’s track^76^. In brief, GPS-enhanced dead-reckoning requires the calculated animal speed, the heading and the GPS location to be known so that the three-dimensional movements of the animal can be calculated between two known positional points^43,44^. Speed was determined during periods when animals are active or moving (i.e., traversing distance), and heading was determined from magnetometer compass heading, which calculated animal direction after correcting for the angle of inclination^43,44,76–78^. GPS fixes were used to determine animal location and to correct for trajectory inaccuracies resulting from magnetometer distortion^43^. Periods of locomotion were identified from accelerometer data using behaviour classifications in DDMT, with animals deemed to be moving when dynamic acceleration (acceleration associated with movement across all axes) exceeded 0.03 g^45,46^. Data from times when animals were moving were then extracted and used for dead reckoning. GPS data were used to correct dead reckoned tracks^76^. Dead reckoned coordinates were exported into QGIS, and converted to a shapefile, where ‘dead reckoned distance’ travelled per night was calculated using the $length command in the field calculator^79^.

#### Statistical analyses

Statistical analyses were performed using R version 1.4.1^80^. Kruskal–Wallis tests were used to determine whether the different home range calculation methods (95% MCP, 95% LoCoH, 95% AKDE) provided differing results and whether nightly distance estimates determined using either just the GPS data or the GPS-enhanced dead-reckoned data differed. To investigate the effects of TVR on badger home range sizes, three separate general linear fixed effect models were undertaken. These were used to investigate: (1) whether home range size using 95% AKDE varied prior to (i.e., for 2014) and during (from 2015 to 2017) TVR; (2) whether ‘core’ home range size measured as 50% AKDE varied prior to and during TVR; and (3) whether home range utilisation (the number of 50% AKDEs) varied prior to and during TVR (Table 1). To examine whether nightly distances travelled differed before and during TVR, two general linear mixed effect models were used. These investigated: (1) whether GPS and GPS-enhanced dead-reckoned distances travelled differed with year, and (2) whether GPS and GPS-enhanced dead-reckoned distances travelled varied with the number of days since animals were trapped. The independent variables included in each model were ‘year’ (four levels: 2014, 2015, 2016 and 2017), ‘night since trapping’ (in days, ranging from one to 19), ‘number of GPS fixes per night’ (ranging from one to eight), life-history characteristics ‘sex’, ‘body mass’ (kg), ‘body length’ (cm), ‘mean daily ambient ‘temperature’, ‘total daily rainfall’, and ‘season’ (four levels: Summer (June to August), Autumn (September to November), Winter (December to February), Spring (March to May)). The distribution of the dependent variables (95% AKDE, 50% AKDE, GPS distance and dead reckoned distance) were non-normal and therefore they were transformed to align with the assumptions of a GLM (generalised linear model, for home range analysis) and GLMM (generalised linear mixed-effects model, for nightly distance travelled analysis) (Table S1)^81^. A two-way interaction between sex and year was included to determine if the effects of TVR varied with sex. Badger identity was included as a random effect in the mixed models.

**Table 1:** General Linear Model (GLM) and General Linear Mixed Model (GLMM) selection. Summaries of selected models for each with the AICc-best fit model are shown below.

Model	Response	Model Type	Random effect	Fixed effects	Description	AIC	Marginal R^2^	Conditional R^2^	*df*	LogLik	AICc	ΔAIC	Weight	Fixed Effect	Chi^2^	*p*
1	95% AKDE	GLM	NA	Null	Final	59.448			2	− 18.79	42.02	0.00	0.44	Null	0.0515449	0.048
2	50% AKDE	GLM	NA	Null	Final	− 19.719			2	15.27	− 26.10	0.00	0.42	Null	4.72E−19	< 0.001
3	Number of 50% AKDE	GLM	NA	Sex	Final	1.4278			3	6.84	− 6.68	0.00	0.21	Sex	1.57E−14	0.017
4	GPS Distance	GLMM	Badger ID	Night + Season + GPS Fix	Final	3521.8	0.0360069	0.2656167	21	− 58.35	165.12	0.00	0.92	Night	58.602	< 0.001
														Season	56.328	< 0.001
														GPS Fix	605.94	< 0.001
5	DR Distance	GLMM	Badger ID	Night + GPS Fix	Final	347.4	0	0.2536522	20	− 155.27	355.76	0.00	0.93	Night	4.791	0.029
														GPS Fix	2.889	0.08919

GLMMs were fitted using the package lme4^81^. Model selection was conducted using the package MuMin, in which global models were simplified using the “dredge” function^82^. The best fit model was selected as the model with the lowest Akaike Information Criteria for small sample sizes (AICc) and the highest weight. Model residuals were checked for normal distribution. Where the assumption of normality was not met, data were transformed (log and square root), and model residuals were re-examined for normality. Model fit was checked using likelihood ratio tests. This established whether there was a significant difference between hierarchical models, with results presented as Chi^2^ values and Probability values (*p* values). Probability values of less than five percent (*p* < 0.05) were interpreted as being statistically significant.

### Ethics approval

This research operated under the Animals (Scientific Procedures) Act 1986 (as amended), ‘ASPA’. The ASPA licences were issued to Department of Agriculture, Environment and Rural Affairs (DAERA) by the Department of Health, Social Services and Public Safety (DHSSPS) in Northern Ireland (Project Licence Numbers 2767 and 2872). Licences were also obtained from Northern Ireland Environment Agency (NIEA) to allow the capture, sampling, collaring and removal of badgers. All methods were performed in accordance with the relevant guidelines and regulations.

## Results

The mean mass of badgers was 9.07 ± 0.71 kg (n = 38 range = 8.07–11.80 kg) with a mean body size of 83.29 ± 2.92 cm (range = 76.0–91.0 cm) and mean head circumference of 27.90 ± 1.60 cm (range = 25.5–33.0 cm). GPS fixes were transmitted for a mean of 78 ± 1.18 days post release (range = 55–273 days). Collared badgers were not sexually dimorphic for body mass (male mean = 9.66 ± 0.95 kg, n = 21, female mean = 8.68 ± 0.39 kg, n = 17), body size (male mean = 84.71 ± 3.09 cm, female mean = 82.23 ± 2.71 cm) or head circumference (male mean = 28.59 ± 1.62 cm, female mean = 27.30 ± 1.61 cm). An average of 6.58 ± 3.47 days (range = 1–17 days) of DD data were recorded before badgers were recaptured.

### Yearly variation in home range size

**Figure 2:**
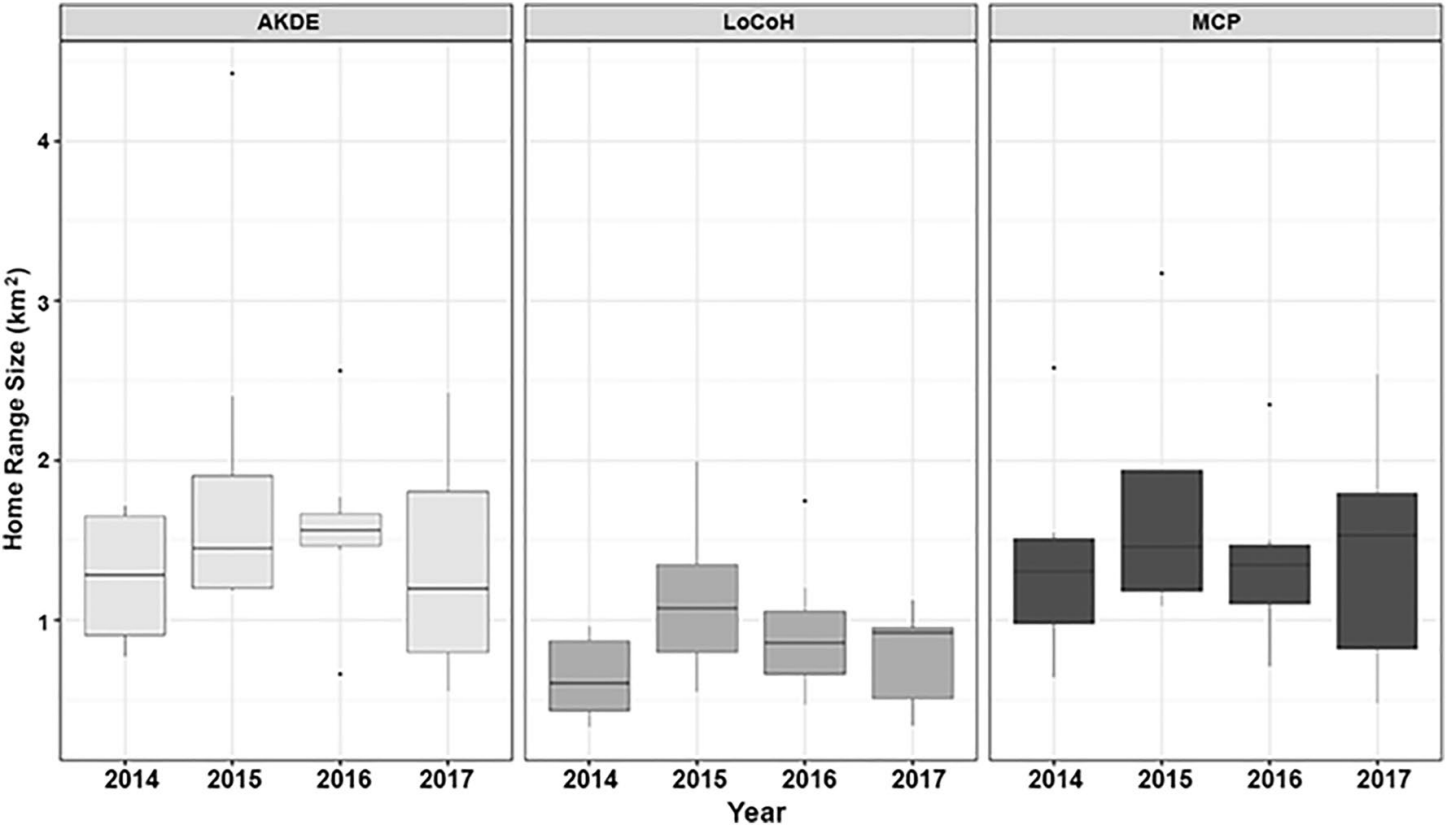
Comparison of home range sizes for each year of the study using three different home range size estimation methods: 95% Home Range Estimators (autocorrelated kernel density estimates (AKDE), local convex hulls (LoCoH) and minimum convex polygons (MCP), measured in square kilometres [km^2^]). The median is shown as a black horizontal line, with upper and lower quartiles (mean point between maximum/minimum data points and the median) at the top/bottom of the bar. Whiskers (vertical black lines) denote lower and upper 25% of data values. Black points denote outliers (data points 1.5 times lower/higher than the interquartile range).

Badger home ranges that were measured using the GPS data and calculated using 95% MCP, 95% LoCoH, 95% AKDE and 50% AKDE provided mean home range estimates of 1.28 ± 0.62 km^2^, 0.83 ± 0.41 km^2^, 1.56 ± 0.62 km^2^, and 0.39 ± 0.62 km^2^, respectively. The 95% home range estimators of these different methods were significantly different (χ^2^ = 17.33, df = 2, *p* < 0.001), with LoCoH method providing smaller estimates than either MCP or AKDE (Fig. 2).

**Figure 3:**
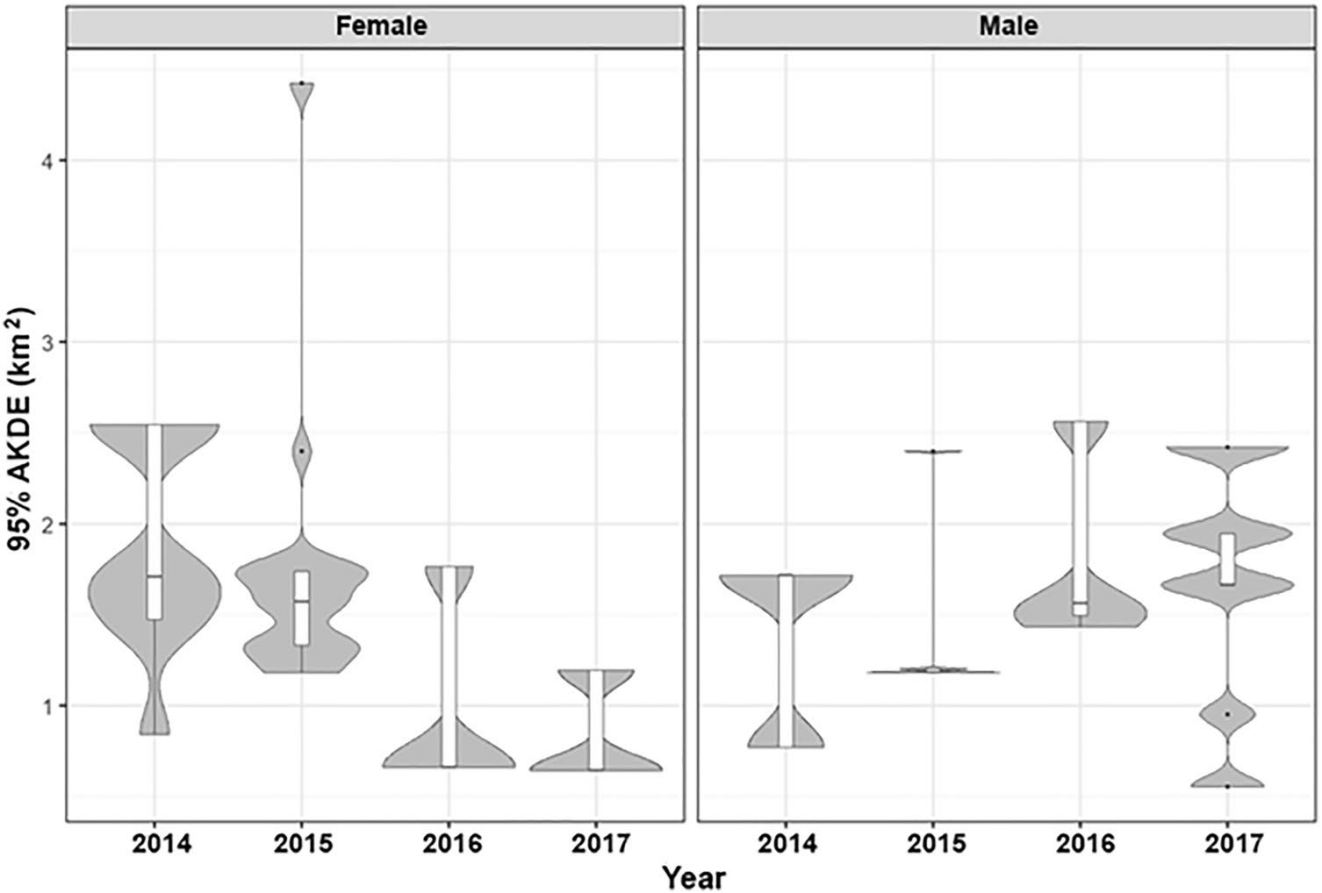
95% autocorrelated kernel density estimate (95% AKDE, km^2^) of female (left) and male (right) badgers for the different years of the study (2014–2017). The median is shown as a black horizontal line, with upper and lower quartiles (mean point between maximum/minimum data points and the median) at the top/bottom of the bar. Whiskers (vertical black lines) denote lower and upper 25% of data values. The density distribution is shown as grey shading.

A selection of the final models, based on lowest AIC value, is shown in Table 1. For the full models, see supplementary information (Table S1). Home range sizes, as determined by 95% AKDE, did not vary significantly during the different years of the study, (F_3686, 3683_ = 0.07, df = 27, *p* = 0.975) (Fig. 2) nor did they differ with sex (F_3686, 3684_ = 0.18, df = 25, *p* = 0.432) (Fig. 3). Similarly, core home range sizes, as determined by 50% AKDE, did not vary significantly over the different years of the study (F_3686, 3683_ = 0.82, df = 27, *p* = 0.461) or with sex (F_3686, 3684_ = 0.18, df = 25, *p* = 0.715). The best fitting GLM for 95% and 50% AKDE home range size was the null model (AICc values: 42.02 and − 26.10, respectively).

**Figure 4:**
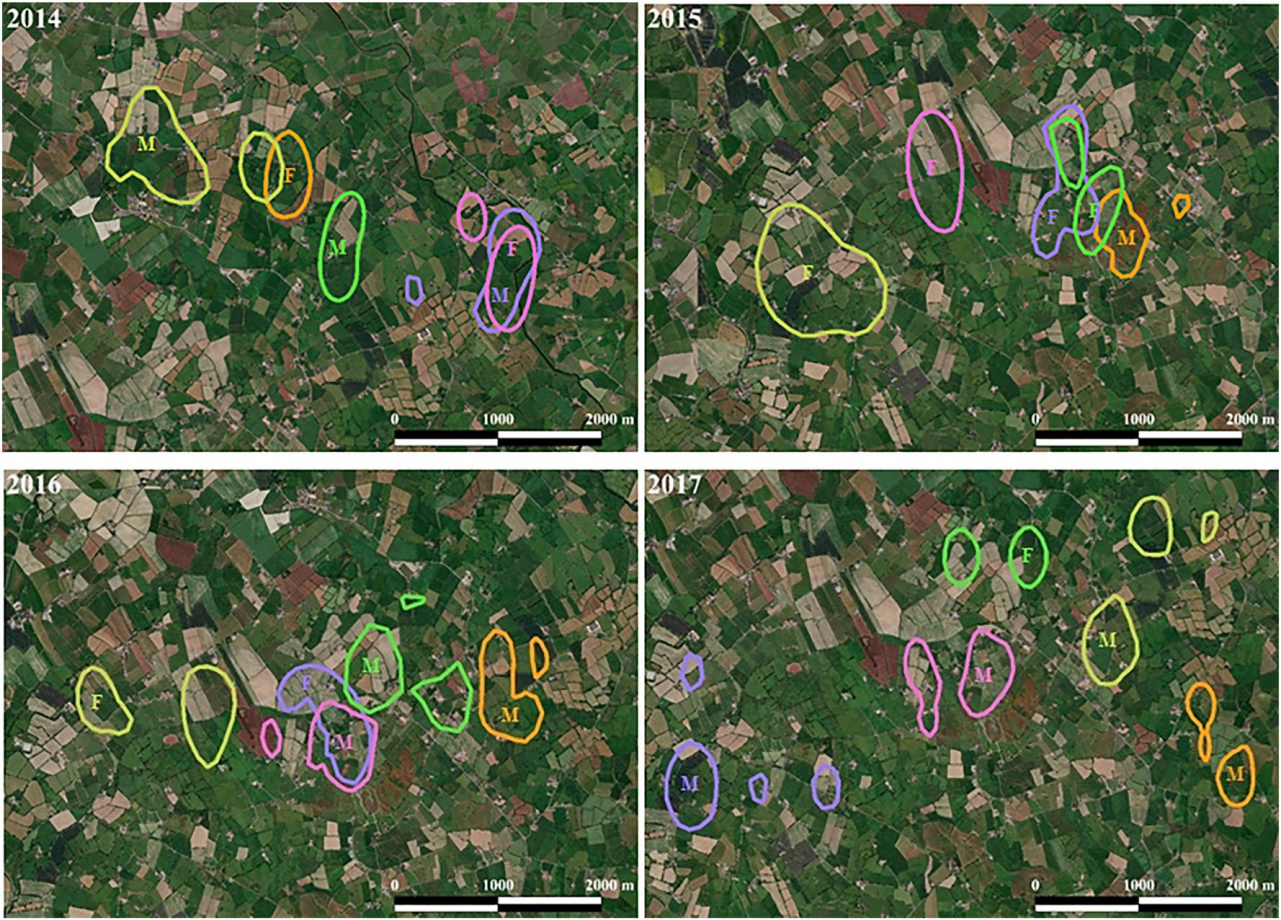
Example of 50% core ranges of 5 badgers for 2014–2017 created using QGIS version 3.8.1 (basemap satellite imagery ERSI World Imagery map) (https://www.qgis.org/en/site/ and https://www.arcgis.com/home/item.html?id=10df2279f9684e4a9f6a7f08febac2a9). Individuals within each year are denoted with a colour overlaid the core region used. Year is denoted in the top left corner of each panel. Sex is denoted by M (male) and F (female).

**Figure 5:**
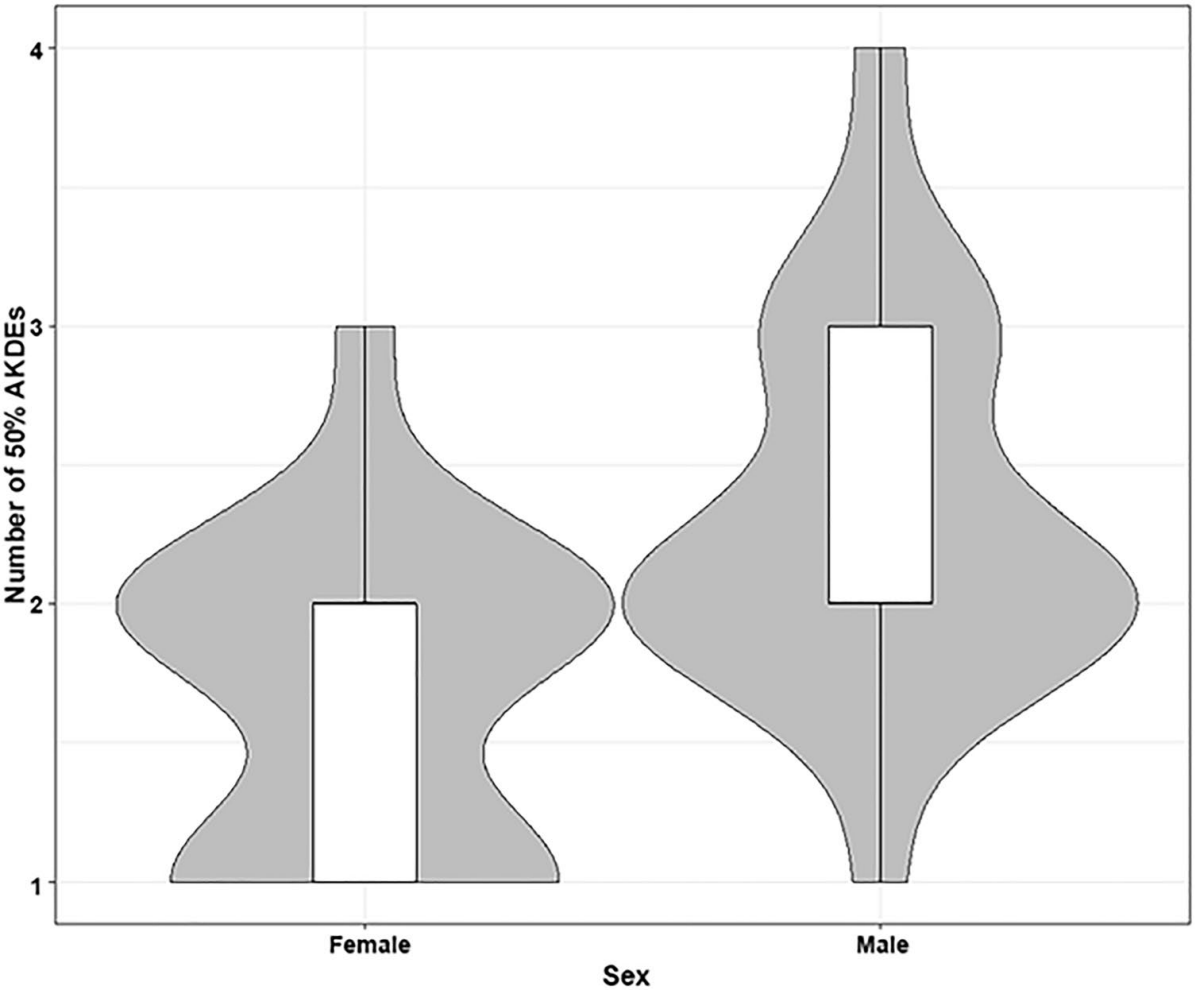
Count of core regions described by the autocorrelated kernel density estimate (number of 50% AKDEs) of female and male badgers. The upper and lower quartiles (mean point between maximum/minimum data points and the median) are shown at the top/bottom of the bar. Whiskers (vertical black lines) denote lower and upper 25% of data values. The density distribution of data is shown with grey shading.

The mean number of core ranges, determined by 50% AKDE, were 1.96 ± 0.63 (range = 1–4), and did not differ with year (F_3693, 3690_ = 2.10, *p* = 0.121) (Fig. 4). The final GLM for the number of 50% AKDE core ranges included sex as a fixed effect (AICc: − 6.68). There was a significant sex-difference in the number of core ranges (F_3693, 3691_ = 7.93, *p* = 0.017) (Fig. 5); male badgers had a greater number of core ranges (mean = 2.31 ± 0.47) than females (mean = 1.66 ± 0.65)*.* The Euclidean distance measured between core areas ranged from 0.36 km to 2.42 km (mean for males = 1.04 ± 0.56, mean for females = 0.24 ± 0.16). There was no relationship between the distance travelled between core areas and year of study (F_3693, 3690_ = 0.73, *p* = 0.539). However, males core areas were further apart than female core areas (F_3693, 3691_ = 5.32, *p* = 0.028) (Fig. 5).

### Nightly distances travelled

The mean nightly distance travelled for all individuals across the years determined using just the GPS data was 1.95 ± 1.18 km (n = 3646 badger nights). The mean nightly distances travelled calculated using GPS-enhanced dead-reckoned data was 3.31 ± 2.64 km (n = 210 badger nights), which was significantly greater than the value calculated using just the GPS data (mean increase = 1.14 km, χ^2^ = 49.04, df = 1, *p* < 0.001). The final GLMM model for GPS nightly distance travelled included night since trapping, season of capture and number of GPS fixes per night as fixed effects (AICc: 165.12). The nightly distance travelled was related to the number of GPS fixes recorded on a particular night (mean number of fixes = 6, χ^2^ = 614.37, df = 1, *p* < 0.001, see Supplementary Table S2), with the calculated distance travelled increasing with the number of fixes obtained.

**Figure 6:**
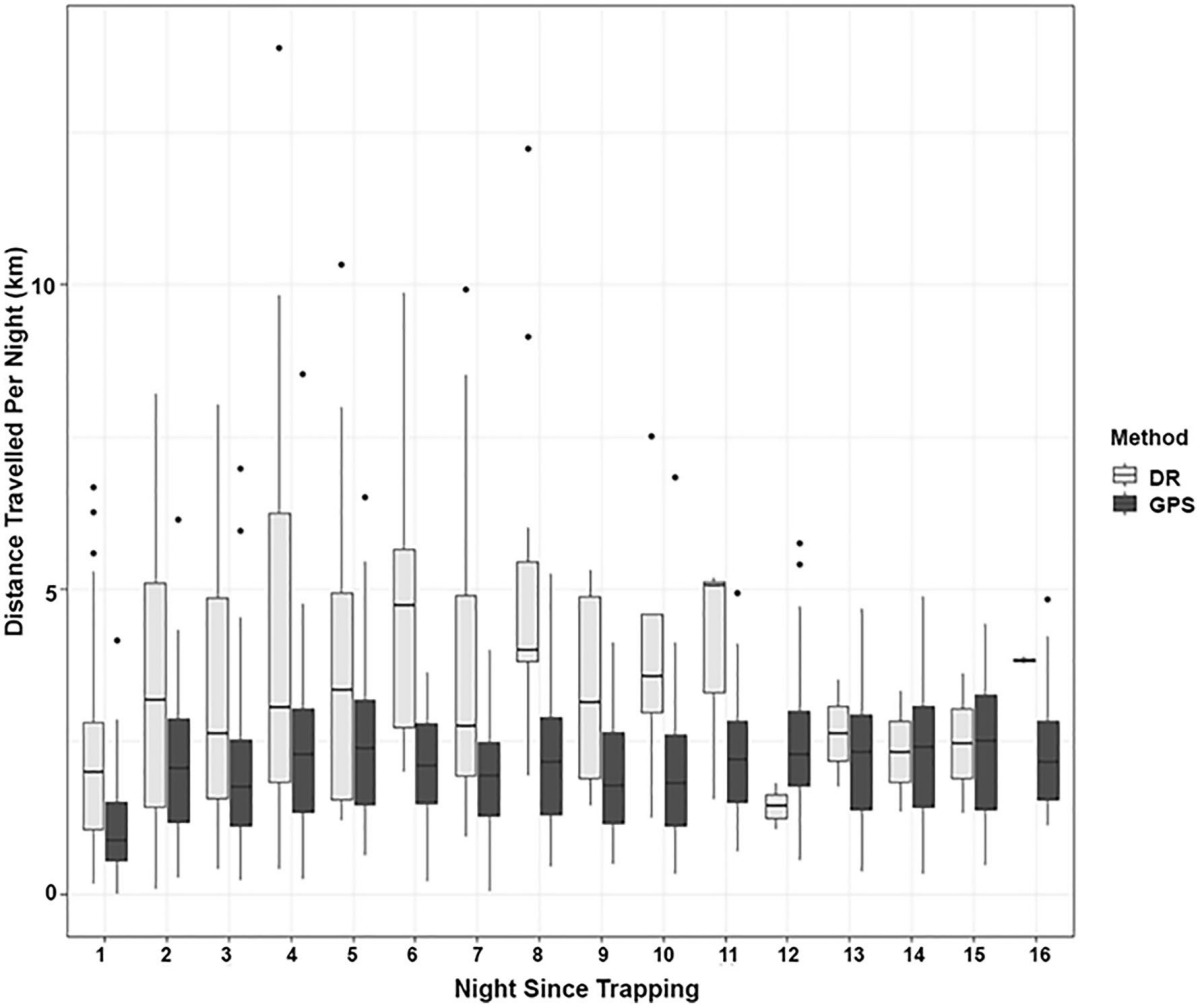
GPS (dark bars) and GPS-enhanced dead-reckoned (light bars) distances travelled per night (km) for 16 nights post trapping. The median is shown as a black horizontal line, with upper and lower quartiles (mean point between maximum/minimum data points and the median) at the top/bottom of the bar. Whiskers (vertical black lines) denote lower and upper 25% of data values. Black points denote outliers (data points 1.5 times lower/higher than the interquartile range).

**Figure 7:**
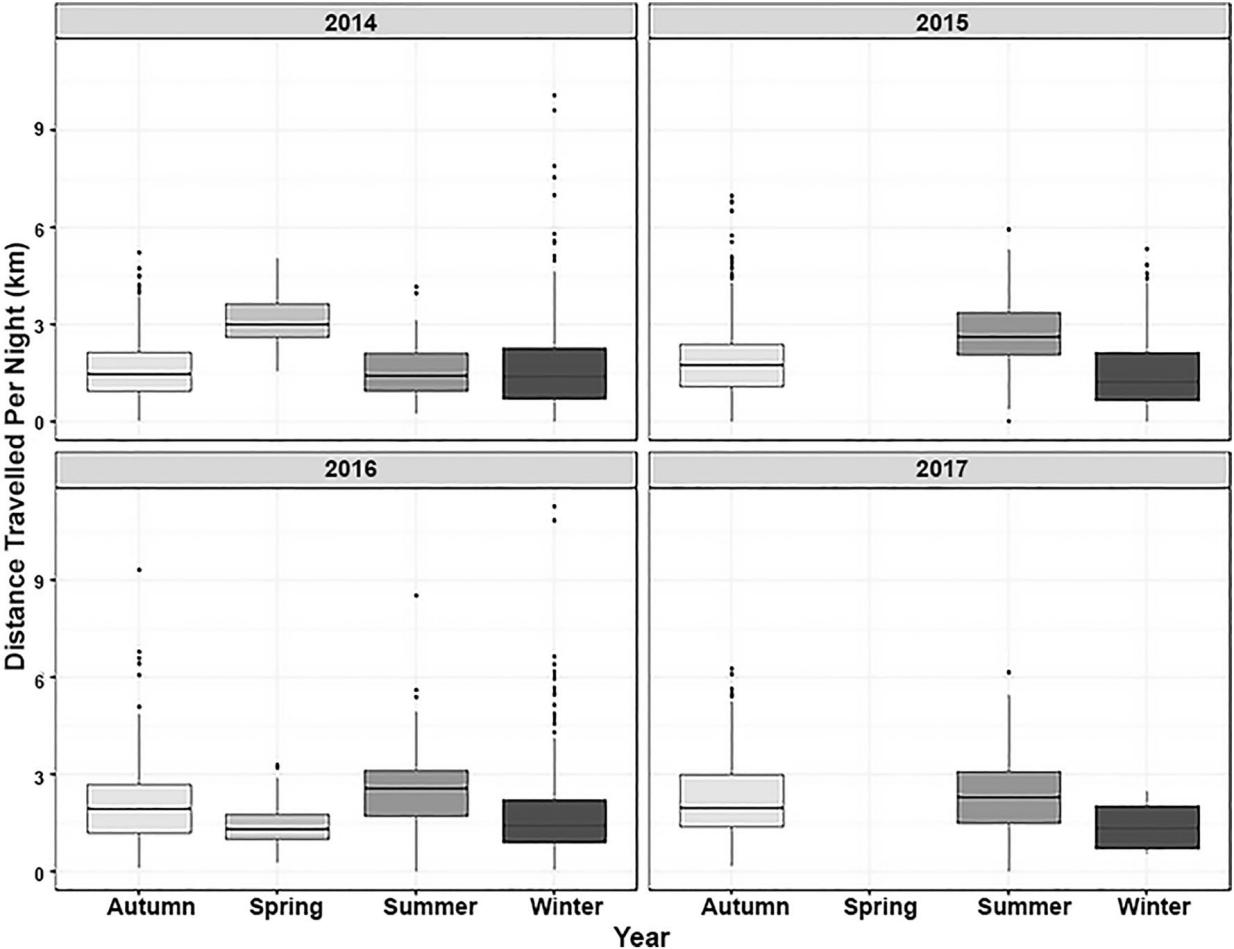
GPS distance travelled per night across seasons for Summer, Autumn, Winter and Spring (where data are available) each year. The median is shown as a black horizontal line, with upper and lower quartiles (mean point between maximum/minimum data points and the median) at the top/bottom of the bar. Whiskers (vertical black lines) denote lower and upper 25% of data values. Black points denote outliers (data points 1.5 times lower/higher than the interquartile range).

**Figure 8:**
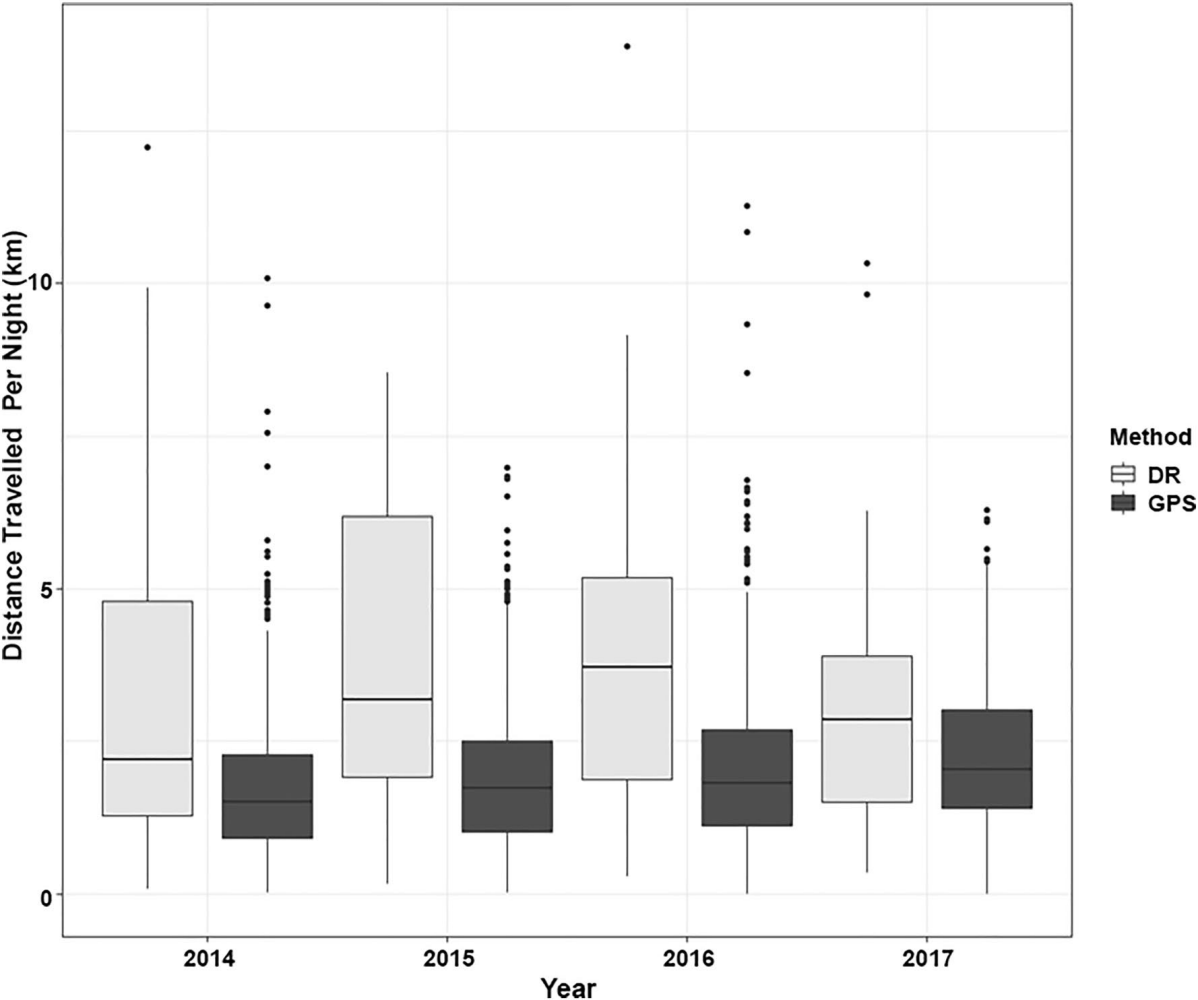
Mean distance travelled per night (km) as calculated using just the GPS data (‘GPS’), dark bars, or the GPS-enhanced dead-reckoned data (‘DR’), light bars, as a function of year (2014–2017). The median is shown as a black horizontal line, with upper and lower quartiles (mean point between maximum/minimum data points and the median) at the top/bottom of the bar. Whiskers (vertical black lines) denote lower and upper 25% of data values. Black points denote outliers (data points 1.5 times lower/higher than the interquartile range).

The distances travelled per night using just the GPS data varied with day post capture. Distances travelled per night gradually increased following capture, with values being lower during the four nights immediately following capture (1.95 km/night) (χ^2^ = 58.60, df = 1, *p* < 0.001, Fig. 6). The calculated distances travelled using the GPS data also varied with season, with shorter distances travelled during winter compared to summer (χ^2^ = 56.32, df = 3, *p* < 0.001, Fig. 7). When the GPS-enhanced dead-reckoned data were examined, the nightly distances travelled were also noted to differ with day since capture. Observed distances travelled were less on night 1, and subsequently increased until night 8 (Fig. 6, χ^2^ = 4.79, df = 1, *p* = 0.029). This model included night since capture and number of GPS fixes as fixed effects (AICc: 355.76). Nightly rainfall (mm) did not have a significant effect on the distances travelled per night (χ^2^ = 0.15, df = 1, *p* = 0.694), nor did badger sex (χ^2^ = 1.34, df = 2, *p* = 0.511). In addition, nightly distances travelled did not differ between years when just the GPS data (χ^2^ = 4.25, df = 3, *p* = 0.235) were examined, or the GPS-enhanced dead-reckoned data were examined (χ^2^ = 2.96, df = 3, *p* = 0.397; Fig. 8).

## Discussion

Studies that have examined badger movement and dispersal, bTB infection, and risks of disease transfer to cattle, have focused on high-density badger populations^14,24,37,72^. For example, in southwest England, badger density can be high (up to 25 badgers per km^2^)^14–16,72^, with 5.8—8.8 individuals per social group^83,84^). In these populations, the social organisation and territorial behaviour adopted by individuals are usually interpreted as a response to the dispersion of resources such as food^85^, denning or sett sites^83^ and/or mating opportunities^86^. However, when badgers are removed from the population, social organisation becomes disrupted and the remaining individuals disperse to acquire increased resources^13,24,30^. In contrast, the removal of badgers from locations in Ireland has not been associated with similar apparent increases in badger movement or effects on bTB prevalence in cattle^23,25^. Badger population density and social group size are typically lower in Ireland, (e.g., 1.9 individuals per km^2^, with 2–6 individuals per social group)^14,87^. At this density, badgers have been observed to engage in inter-group movements more frequently which is thought to facilitate breeding and foraging^47^. It has therefore been suggested that at lower population densities, increased inter-group movements and reduced competition for resources lessen the motivation for permanent displacement in response to culling^47,48^. Another corollary of increased inter-group movement is that vaccination of badgers may be particularly effective in reducing transmission when increased contact between social groups exists, which reduces badger-to-badger bTB transmission^88^ and thereby spillover of bTB from badgers to cattle but does not prevent cattle-to-badger transmission. In the current study, we examined the movements and home ranges of badgers in a medium density population in NI prior to and during TVR operations. Removal of bTB-positive individuals and vaccination of individuals that are captured has been suggested as a strategy to reduce the prevalence of bTB in badgers^36^. The current study builds on findings from studies in higher population density areas in England^24^ and assesses the effects of TVR in lower badger population density areas. The mean badger home range size recorded was 1.56 ± 0.62 km^2^ (95% AKDE, up to 2.56 km^2^), which was comparable to observations in the same area 20 years earlier (1.27 km^2^^18^) and did not change during the years of the study, prior to and during TVR. Thus, badger home ranges seem to have been stable for several years^18,89^. There were, however, apparent sex differences in home range use with males utilising core areas that were further apart suggesting which might suggest variation in territorial behaviours^49,90^. By comparison, home range sizes were smaller in higher density badger populations, such as in Cornwall (density = 4.2–6.3 badgers/km^2^, mean home range size = 0.45km^2^^72,91^) and Gloucestershire (density = 7.8–47 badgers/km^2^, mean home range size = 0.25km^2^^15,92^), in southwest England.

Links between food abundance, population density and badger home range size were considered by Kruuk^93^ who suggested that, as food abundance in an area increases, badger density increases and territory sizes decrease^85,94^. Territorial behaviours such as boundary patrolling and fighting are thought to increase as the density of badgers in neighbouring areas increases, although movement and breeding between groups in high-density areas persists^15,49,90,95^. In Ireland, it has been suggested that the population may be at carrying capacity, due to fewer suitable hedgerow and woodland areas that limit sett construction^19,89^. Where badger density is low (< 1 individual per km^2^), territorial behaviour such as latrine marking at boundaries may diminish^96,97^. Byrne et al*.* (2019) found that badgers dispersed farther and more often when population density was low (0.8–1.1/km^2^), compared to individuals from higher population density areas (4.3–11.61/km^2^) from study sites in County Kilkenny^98^. They suggested that within higher density populations, strong territoriality creates a greater energetic cost to regular inter-group movements as individuals may face aggression from neighbouring social groups and consequently ‘social fluidity’ is reduced^97^. By comparison, in medium density populations such as the current study, home range sizes are larger, ranging movement is increased and territories are expected to be ‘looser’ to facilitate breeding and foraging^14,98^. Alternatively, it may be the case that geographic isolation caused by the Irish Sea has enabled different epigenetic pathways to proceed in each country, influencing genes regulating aspects of social behaviour and susceptibility to disease^99^. It is possible that because inter-group movements increase at lower badger densities, there is a reduced motivation for permanent displacement in response to badger removal or culling^47,48^. However, this doesn’t exclude the possibility that badgers could move or disperse farther, even in low density populations, when individuals are removed. In addition, within the current study area, whilst social group overlap did not appear to change following TVR operations extra-group paternity may have increased^100^. This suggests that there may well be subtle effects of TVR that are missed by basic analyses of home range, and more detailed analyses are needed to understand the effects of TVR on badger behaviour.

In the current study, when GPS-enhanced dead-reckoned data were used to elucidate detailed movements^56^, badgers were observed to travel 3.31 ± 2.64 km per night (maximum = 13.88 km). This is compared with shorter calculated distances travelled using just the GPS data (mean = 1.95 ± 1.18 km, maximum = 11.27 km). The latter values were similar to those reported in previous studies in Ireland (1–2 km per night^47,48^), and are greater than those recorded in high-density areas in Southwest England (0.6–1.9 km per night^24,72,74^). Badgers in low density populations in other countries (e.g., Portugal, density = 0.36—0.48 badgers per km^2^) have been reported to travel as far as 17.5 km per night^101^, which can occur within a home range or relate to an inter-group movement^14,48^. Therefore, it is not necessarily the case that an increase in measured badger distance travelled results in an increase in home range size or an increase in the contact between individual badgers (or possibly cattle). The largest nightly distance we recorded was 13.88 km by one male. This occurred four nights post-trapping, and the badger was active but did not venture beyond the boundaries of the 95% AKDE home range. This behaviour could be associated with territorial ranging, either because of trapping causing stress^41,102^ and restricting movement^41^, or an inter-group excursion^47,48,97^ for foraging around the territory boundary. Interesting, weather variables (temperature and mean precipitation) did not significantly influence badger ranging, which contrasts with the findings of previous studies^14,18^. It is possible that the variation in climate during the study period was insufficient to generate changes in badger ranging. Whether or not rainfall affects behaviour is likely to be moderated by background conditions. For example, predictable and frequent rainfall is likely to have less of an effect than infrequent and unpredictable rainfall, such as rain after a two-week drought.

The use of GPS-enhanced dead-reckoning to calculate distances travelled resulted in greater estimates than did the use of GPS data alone (mean increase = 34.41%; 1.14 km per night)^56^. Given the increased precision of movements determined using GPS-enhanced dead-reckoned data^76^, this method of determining geographical movements should aid in the understanding of inter-group movements in badgers. We suggest that previous reported badger ranging behaviour in low to medium density populations could have been underestimated and GPS-enhanced dead-reckoning may be useful as an additional method to detect potential dispersal events^48,56^.

The change in badger home range size, inter-group movement and bTB prevalence following culling, known as the “perturbation effect”, has been suggested to occur in several localities in England^13,24,30,34^. However, this has not been reported in lower density populations in Ireland^23,25,47^. We did not find changes in home range size or nightly distances travelled during TVR operations^51,52^. The lack of apparent effects on badger movements following TVR does however need to be interpreted with caution. It is currently unclear whether the reason that badgers do not appear to change in distances travelled or home range following TVR is due to the lack of effects of removals, or because of any effects of vaccination on behaviour. Indeed, it is not possible to separate the effects of vaccination from the effects of removing potentially infectious badgers on any apparent decrease in bTB levels noted within the badger population^51^. Other confounding factors might concern the efficacy of the field DPP VetTB test. The number of false negatives (comparing negative tests in the field with subsequent positive laboratory serum IFN-γ tests) was small, at 1.8%, but the number of false positives (comparing positive tests in the field with subsequent negative laboratory tests) was large, at 54%^50^, indicating that approximately half of the badgers that were euthanised were later found to be bTB negative. In addition, potential concerns might be raised regarding seroconversion of the BCG vaccine, and subsequent (next year) field DPP test-positive results, especially as faint lines could be interpreted as bTB positive, although this has been shown to be unlikely to occur from a single vaccination^57^. Lastly, the current research only monitored adults and individuals that were suitable to fit a collar^52^, however, younger, or perhaps badgers with small heads (and hence not collared) might be individuals that are affected by TVR, but these individuals were not monitored. Hence, there are ethical concerns (euthanising healthy badgers) as well as experimental concerns such as increasing the likelihood of a perturbation effect (by removing more badgers than is necessary), potential DPP false-positive results caused by vaccination and lack of monitoring of all members of the population that need to be considered before conclusions are drawn about the effects of TVR on badger movement. Badgers were, however, affected by being trapped and were observed to move greater distances up to 8 days post trapping (mean = 3.47 ± 2.72 km), after which nightly distances travelled decreased (mean = 2.33 ± 1.85 km). Thereafter, distances travelled were similar to those observed in previous studies in Ireland^14,47,48^. While increased distances travelled have been associated with visits to foraging patches and inter-group movements^47^, in this case, an increase in movement post trapping may be more likely to be a response to the stress and possibly increased hunger caused by overnight trapping^41,102^, even though badgers gained some nutrition by eating the peanut bait. This is important as increases in distances travelled and extra-territorial excursions have been shown to relate to positive disease status^33^, either as a result of inter-group contact facilitating transmission^47^, bTB progression altering ranging behaviour^37^ or from the physiological stress of trapping reducing immuno-competence and thereby increasing disease susceptibility^15^. Hence, these increases in ranging may contribute to the spread of bTB within badgers across a landscape^27,28^ and suggest that interference by humans, for example by trapping or disturbances at the sett, might influence bTB transmission. Therefore, it may be beneficial to utilise solutions such as oral vaccination and increased biosecurity to limit disturbance to badgers^72^, and thereby limit the likelihood of bTB transmission^72,74,103–105^.

## Conclusions

An increase in badger ranging following culling has been observed in several high-density populations in England^13,24,30,34^. This has been associated with increased bTB prevalence in surrounding cattle herds^26^. However, the perturbation effect has not been observed in areas with lower badger density, such as Ireland^23,25^. Just why potential differences in behaviour and ranging exist between British and Irish badger populations remains unclear, but it is likely to include many facets, such as habitat suitability, carrying capacity and anthropogenic disturbance^16^. The current study indicates that longer-range movements are likely to be more frequent in lower density populations, and similar to that previously reported in Ireland, analyses of GPS data indicated that TVR operations may not result in changes to badger home range^13,24,26,44,52^. Whether or not TVR alters more subtle aspects of badger behaviour^24,34^ remains to be seen. However, it would be better to utilise less intrusive solutions such as oral vaccination of badgers and/or cattle as well as increased biosecurity to limit the likelihood of bTB transmission^72,74,103–105^.

## Supplementary Information

Supplementary Table S1.

Supplementary Table S2.

## Data Availability

The datasets used and/or analysed during the current study are available from the corresponding author on reasonable request.

## Abbreviations

AKDE: Autocorrelated kernel density estimate
BCG: Bacillus Calmette–Guérin
bTB: Bovine tuberculosis
DAERA: Department of Agriculture, Environment and Rural Affairs
DR: Dead-reckoning
GPS: Global positioning system
MCP: Minimum convex polygon
NI: Northern Ireland
ROI: Republic of Ireland
TVR: Test, Vaccinate or Remove
UK: United Kingdom
