# Supplementary material for: Impact of test, vaccinate and remove protocol on home ranges and nightly movements of badgers in a medium density population
Source: Sci Rep. 2023 Feb 14;13:2592. doi: 10.1038/s41598-023-28620-1 (PMC9929337; doi:10.1038/s41598-023-28620-1)
Supplement: Supplementary file 1 — Supplementary Table S1. [file 41598_2023_28620_MOESM1_ESM.pdf]

Table S1: Generalised linear effect model and generalised linear mixed effect model selection for home range analysis (95% and 50% autocorrelated kernel density estimates) and nightly distances travelled (GPS and dead reckoned estimates). Significant results are in bold.

Response	Model Type	Random effect	Fixed effects	Terms removed	Description	*df*	LogLik	AICc	ΔAIC	Weight	Fixed Effect	Chi^2	*p*
95% AKDE (log)	GLM	NA	Year * Sex + Sex * Mass * Body Size * Head Size		Global	23	9.27	211.45	169.44	0.00			
			Sex * Mass * Body Size + Sex * Head Size	Year	Intermediate	17	-12.50	110.00	67.97	0.00	Year	0.0011869	0.9994
			Year + Mass * Body Size * Head Size	Sex	Intermediate	9	-16.27	59.54	17.52	0.00	Sex	0.0040411	0.4329
			**Null**	**Null Retained**	**Final**	**2**	**-18.79**	**42.02**	**0.00**	**0.44**	**Null**	**0.0515449**	**0.04883**
50% AKDE (sqrt)	GLM	NA	Year * Sex + Sex * Mass * Body Size * Head Size		Global	23	45.24	139.50	165.61	0.00			
			Sex * Mass * Body Size * Head Size	Year	Intermediate	17	23.77	37.46	63.56	0.00	Year	4.64E-16	0.4617
			Year + Mass * Body Size * Head Size	Sex	Intermediate	9	18.86	-10.72	15.38	0.00	Sex	4.42E-15	0.7152
			**Null**	**Null Retained**	**Final**	**2**	**15.27**	**-26.10**	**0.00**	**0.42**	**Null**	**4.72E-19**	**0.0006088**
Number of 50% AKDE (sqrt)	GLM	NA	Year * Sex + Sex * Mass * Body Size * Head Size		Global	23	48.35	225.31	226.17	0.00			
			Sex * Mass * Body Size + Sex * Head Size	Year	Intermediate	17	13.68	67.84	68.70	0.00	Year	5.28E-14	0.1217
			Sex * Body Size + Sex * Head Size	Mass	Intermediate	9	10.56	6.88	13.56	0.02	Mass	1.67E-13	0.1312
			Sex	**Sex Retained**	**Final**	**3**	**6.84**	**-6.68**	**0.00**	**0.21**	**Sex**	**1.57E-14**	**0.01749**
GPS Distance (sqrt)	GLMM	Badger ID	Sex * Year * Night + Sex * Body Size * Mass + Sex * Season + Temperature + Rainfall + GPS Fix		Global	87	-12.98	396.27	231.15	0.00			
			Sex * Night + Sex * Body Size * Mass + Sex * Season + Temperature + Rainfall + GPS Fix	Year	Intermediate	41	-48.01	205.80	40.68	0.00	Year	8.17E+00	0.235
			Sex * Night + Sex * Body Size + Sex * Season + Temperature + Rainfall + GPS Fix	Mass	Intermediate	37	-50.02	196.02	30.89	0.00	Mass	6.70E-01	0.413
			Sex * Night + Sex * Body Size + Sex * Season + Temperature + GPS Fix	Rainfall	Intermediate	36	-50.18	193.01	27.89	0.00	Rainfall	*5.397*	0.02017
			Night + Season + Body Size + Temperature + GPS Fix	Sex	Intermediate	23	-58.16	170.10	4.98	0.08	Sex	1.3402	0.5116
			**Night + Season + GPS Fix Number**	**Night, Season and GPS Fix Number Retained**	**Final**	**21**	**-58.35**	**165.12**	**0.00**	**0.92**	**Night**	**58.602**	**1.93E-14**
											**Season**	**56.328**	**3.58E-12**
											**GPS Fix**	**614.37**	**2.20E-16**
DR Distance (sqrt)	GLMM	Badger ID	Year * Sex * Night + Sex * Body Size * Mass + Temperature + Rainfall + GPS Fix		Global	86	-114.66	558.83	203.07	0.00			
			Year * Night + Body Size * Mass + Temperature + Rainfall + GPS Fix	Sex	Intermediate	52	-129.84	406.42	50.66	0.00	Sex	2.2189	0.3297
			Year * Night + Body Size * Mass + Temperature	Rainfall	Intermediate	51	-130.21	403.21	47.45	0.00	Rainfall	0.1546	0.6942
			Night + Body Size * Mass + Temperature	Year	Intermediate	24	-152.65	360.93	5.17	0.07	Year	7.0997	0.3976
			Night + GPS Fix	**Night and GPS Fix Retained**	**Final**	**20**	**-155.27**	**355.76**	**0.00**	**0.93**	**Night**	**4.791**	**0.02861**
											**GPS Fix**	**2.889**	**0.08919**
