# Supplementary material for: Impact of test, vaccinate and remove protocol on home ranges and nightly movements of badgers in a medium density population
Source: Sci Rep. 2023 Feb 14;13:2592. doi: 10.1038/s41598-023-28620-1 (PMC9929337; doi:10.1038/s41598-023-28620-1)
Supplement: Supplementary file 2 — Supplementary Table S2. [file 41598_2023_28620_MOESM2_ESM.pdf]

Table S2: Mean GPS and dead reckoned distance travelled (km) per number of GPS fixes available per night

Number of GPS Fixes Per Night	Mean GPS Distance Travelled (km)	Standard Deviation	Mean DR Distance Travelled (km)	Standard Deviation
1	0.519	0.569	0.578	0.293
2	0.821	0.615	1.24	1.31
3	1.22	0.708	1.30	0.528
4	1.49	0.928	3.05	2.49
5	1.78	1.18	3.42	3.14
6	2.01	1.07	2.99	2.26
7	2.32	1.13	3.71	2.70
8	2.40	1.13	3.85	2.82
